# Social Observation Increases Functional Segregation between MPFC Subregions Predicting Prosocial Consumer Decisions

**DOI:** 10.1038/s41598-018-21449-z

**Published:** 2018-02-20

**Authors:** Daehyun Jung, Sunhae Sul, Minwoo Lee, Hackjin Kim

**Affiliations:** 1 Laboratory of Social and Decision Neuroscience, Korea University, Seoul, Republic of Korea; 2 Department of Brain and Cognitive Engineering, Korea University, Seoul, Republic of Korea; 3 Department of Psychology, Korea University, Seoul, Republic of Korea; 4 Department of Psychology, Pusan National University, Busan, Republic of Korea; 5 Present Address: Department of Anthropology, Emory University, 201 Dowman Drive, Atlanta, GA 30322 USA

## Abstract

Although it is now well documented that observation by others can be a powerful elicitor of prosocial behaviour, the underlying neural mechanism is yet to be explored. In the present fMRI study, we replicated the previously reported observer effect in ethical consumption, in that participants were more likely to purchase social products that are sold to support people in need than non-social products when being observed by others. fMRI data revealed that the anterior cingulate cortex (ACC) and the dorsomedial prefrontal cortex (dmPFC) encoded subject-specific value parameters of purchase decisions for social and non-social products, respectively, under social observation. The ACC showed strong functional coupling with the amygdala and the anterior insula when participants in the observation condition were making purchases of social versus non-social products. Finally, ventromedial prefrontal cortex (vmPFC) activity predicted faster reaction time and increased prosocial behavior during decisions to purchase social versus non-social products, regardless of social observation. The present findings suggest that subregions of the mPFC, namely the dmPFC, ACC, and vmPFC, are hierarchically organized to encode different levels of decision values from the value of context-sensitive reputation to that of internalized prosociality.

## Introduction

People often pay extra money to purchase environment-friendly goods or fair-trade products. This phenomenon, often referred to as “ethical consumption”^1^, is puzzling when viewed from a purely economic standpoint: Why would people invest their economic resources into seemingly extraneous values such as prosociality?

One plausible explanation comes from the “costly signaling theory,” which views altruistic behavior as a signal of willingness and ability to help others^2,3^. Here, a concern for one’s reputation is the key motivation for altruistic behavior, since positive social standing, once established, will ultimately pay off in repeated social interactions^4,5^.

Consistent with the notion of altruism as a signalling strategy, abundant evidence has shown that people tend to demonstrate increased prosocial tendency when their reputation concern is made salient either by social observation or by subtle surveillance cues^6^. For example, consumers are more likely to favor ethical products over more luxurious alternatives when their decisions are made in public versus in a personal context^7,8^. The presence of others is also known to promote generosity in economic games^9^ and charitable giving^10,11^, as well as positive self-appraisal^12^.

Despite the close entanglement between reputation concern and prosocial behavior, however, a specific neural mechanism mediating this relationship remains to be identified. One potentially important brain structure that underlies the interaction between reputation concern and prosocial behaviors is the medial prefrontal cortex (MPFC), which comprises multiple functionally dissociable subregions^13^. The anterior cingulate cortex (ACC), for example, becomes particularly active in the presence of observers^14,15^, potentially guiding one’s reputation promotion in the face of evaluative others^16,17^.

A developing body of evidence also suggests functionally distinct contributions of the ventromedial (vmPFC) and the dorsomedial prefrontal cortex (dmPFC) to social behaviors. Previous studies have postulated that vmPFC activity reflects automatic/intuitive processes related to “first-person” information^18–20^, whereas the dmPFC is more involved in a deliberative/controlled, “third-person” mode of decisions^21–28^. Several studies have also shown that vmPFC activity can be commonly involved in decisions for both self and others, particularly when applying self-simulation to estimate a stranger’s preferences^29^ and when people are fully familiarized with other’s preferences through practices^30^. Similarly, in the specific context of prosociality, the vmPFC seems to encode decision values for highly internalized forms of altruistic behaviors (i.e., internalized prosocial valuation) as in harm-aversion in social dilemma and moral emotions^31–38^, whereas the dmPFC is involved in decisions that are strategically beneficial^22,39,40^.

**Figure 1:**
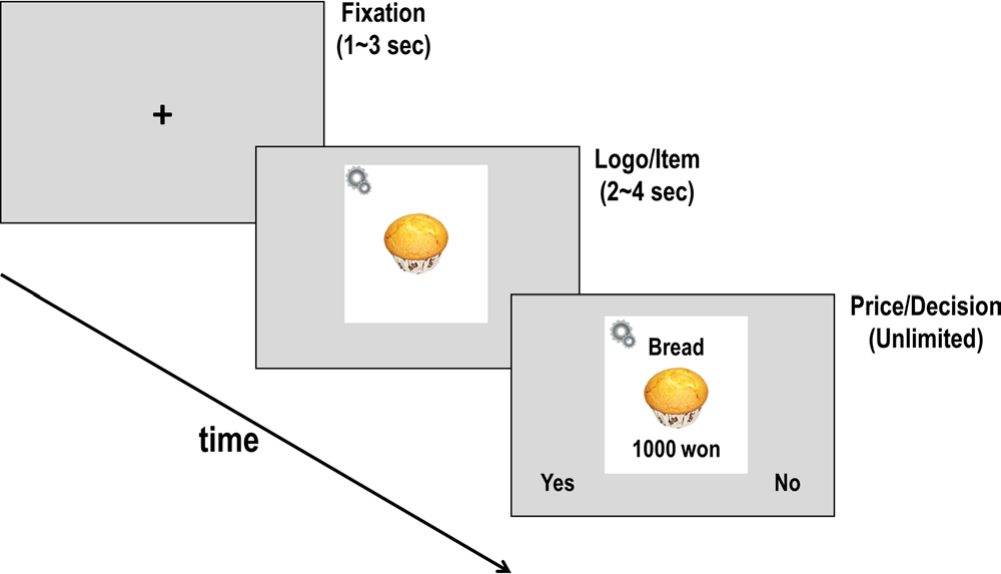
Virtual shopping task. An example of a single trial in the virtual shopping task. Non-social product image (muffin: https://cdn.pixabay.com/photo/2017/04/04/16/34/cake-2201816_960_720.jpg) and brand logo of non-social product image (cogwheel: https://cdn.pixabay.com/photo/2013/07/12/12/30/cogwheel-145804_960_720.png) by Pixabay is licensed under CC0 Creative Commons (https://creativecommons.org/publicdomain/zero/1.0/legalcode).

These findings altogether suggest that the impact of reputation concern on prosocial behaviors could be subserved by systematic patterns of neural activation across differential subregions in the MPFC. In the present study, we adopted a novel “ethical consumption task,” where participants were instructed to make a series of binary purchase decisions on food items at given prices (Fig. 1), and manipulated the level of reputation concern to investigate the neural mechanisms of prosocial behavior in public versus in private.

Prior to the experiment, participants were informed about the definition of ethical consumption: we explained to participants various forms of consumption behavior that promote social value, based on the examples of social enterprise products such as environment-friendly and fair trade products. Then, participants were instructed to make purchasing decisions on social or non-social products at various price levels, and were told that social products were manufactured by social enterprises, whereas non-social products were manufactured by general companies that pursue profit-maximization. Participants in the OBS group were told that two experimenters outside the scanner room would monitor and manually record their responses due to some technical issues^12,14^. Those in the CON group, on the contrary, were assured that their responses would remain undisclosed. In each trial, either social or non-social products were presented at one of seven different price levels: 25%, 50%, 75%, 100%, 125%, 150%, or 175% of the optimal price determined in the BDM experiment (see the Methods for details).

We specifically aimed to reveal functionally unique contributions of the MPFC subregions to prosocial behavior under social observation. We hypothesized that the subject-specific value-related activity in the ACC would increase under social observation, promoting context-dependent prosocial behavior. In addition, we predicted that the vmPFC would be involved in computing internalized values of prosocial choices that are independent of observational context, whereas the dmPFC would be engaged in context-dependent strategic and deliberative decision processes.

## Results

### Behavioral results

#### Purchase decision

**Figure 2:**
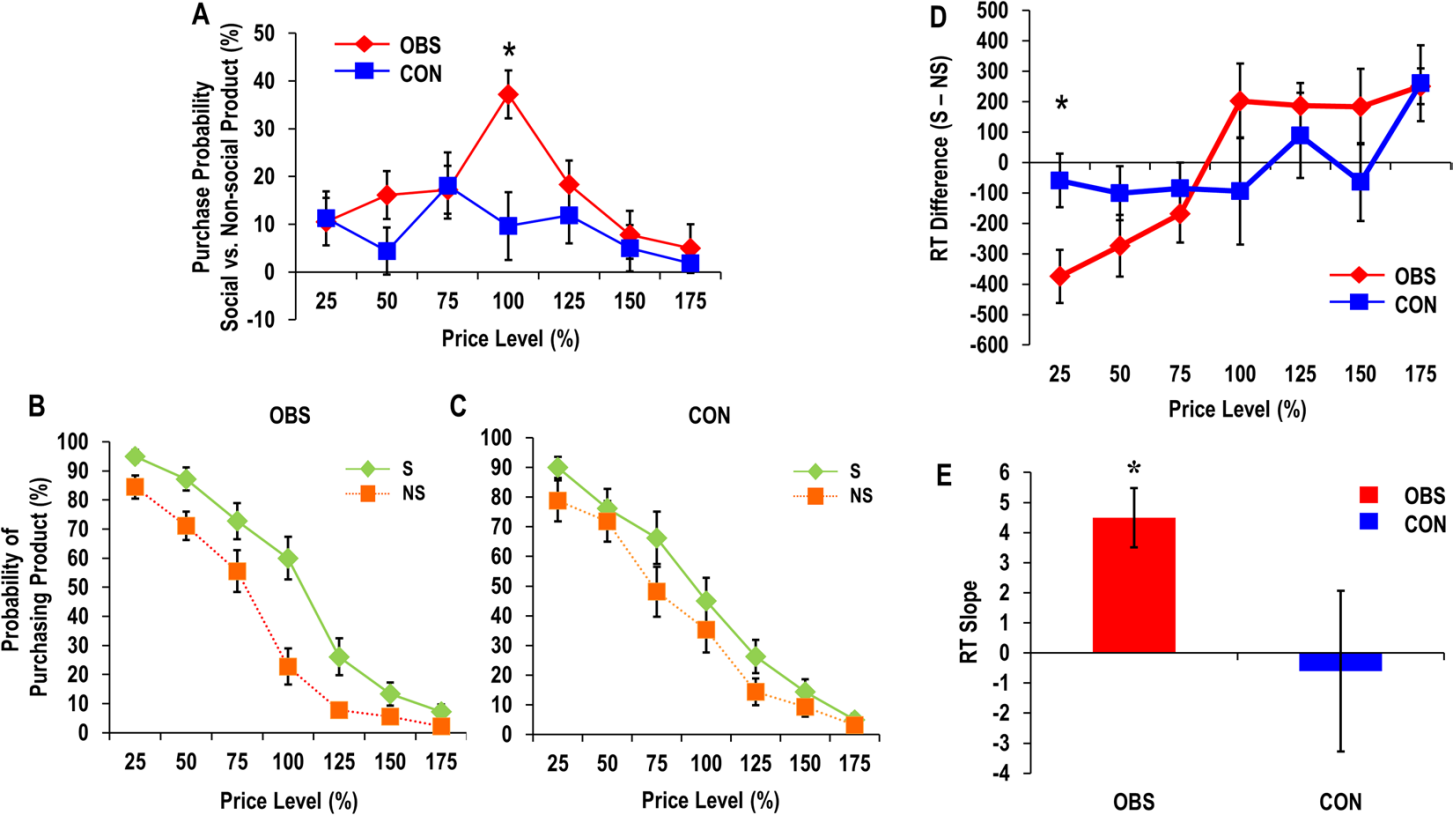
Behavioral Results. (**A**) Differential purchase probability between social (S) and non-social (NS) product conditions as a function of price level in the observation (OBS) and the control (CON) group. Probability of purchasing for social and non-social products in the (**B**) OBS and the (**C**) CON group. (**D**) Differential response time (RT) between social and non-social product conditions as a function of price level in the OBS and the CON group. (**E**) Mean slope of linear regression line best fitted to the differential RT between social and non-social product conditions as a function of price level in the OBS and the CON group. All the error bars in the present manuscript indicate standard errors.

Based on previous findings, we expected that the participants in the observation (OBS) group would purchase social versus non-social products more than the participants in the control (CON) group. To test this hypothesis, we performed a three-way mixed ANOVA with product type (social and non-social product) and price level (25–175%, seven levels altogether) as within-subject factors and group (OBS and CON group) as between-subject factor. Confirming our prediction, this analysis yielded a significant three-way interaction effect on purchase rates (*F*_(6,192)_ = 3.69, *p* < 0.05). We also found the main effects for product type (*F*_(1,32)_ = 25.52, *p* < 0.05) and price level (*F*_(6,192)_ = 189.73, *p* < 0.05), but not for group (*F*_(1,32)_ = 0.14, *p* = 0.71). Post-hoc analyses revealed that the three-way interaction effect was mainly driven by the OBS group showing higher purchase rates for social than non-social products at the 100% price level compared to the CON group (*F*_(1,32)_ = 9.70, *p* < 0.05, Fig. 2A). The reason why the group difference was significant only at one price level (100%) may be because the value curve for the social product moved to the right as a whole, shifting the indifference point of the curve. This then makes the point at which the gap between the two value curves is widest found at the midpoint.

A repeated-measures two-way ANOVA for the OBS group revealed a significant interaction between product type and price level (*F*(6,102) = 10.06, *p* < 0.05), and main effects for product type (*F*(1,17) = 30.75, *p* < 0.05) and price level (*F*(6,102) = 149.40, *p* < 0.05), showing higher probability of purchasing social than non-social products and increasing probability of purchasing as price level decreased (Fig. 2B). A repeated-measures two-way ANOVA for the CON group did not show a significant interaction between product type and price level (*F*(6,90) = 1.89, *p* < 0.13), although main effects for product type (*F*(1,15) = 4.69, *p* < 0.05) and price level (*F*(6,90) = 62.72, *p* < 0.05, Fig. 2C) were significant. In the CON group, there was a significant difference between the social product and non-social product conditions only at the 75% price level (*p* < 0.05), whereas, in the OBS group, the purchasing rates of the social vs. non-social products were significantly greater at all the price levels (all *ps* < 0.05).

#### Reaction time

We predicted faster response times (RT) for purchasing social products especially at low price levels in the OBS group, due to prosocial valuation facilitated by social observation. In terms of RT data, we found a marginally significant three-way interaction effect (*F*_(6,192)_ = 2.63, *p* = 0.057), along with significant main effects for price level (*F*_(6,192)_ = 5.66, *p* < 0.05), but not for group (*F*_(1,32)_ = 0.002 *p* = 0.96) and product type (*F*_(1,__32)_ = 0.02, *p* = 0.89). Post-hoc analyses revealed that this interaction effect was mainly driven by faster decisions made by the OBS group than the CON group at the lower price level (25%) for social versus non-social products (*F*(1,32) = 6.41, *p* < 0.05, Fig. 2D). It is noteworthy that decisions were faster (or slower) for social versus non-social products at lower (or higher) price levels, especially in the OBS group, since this indicates that observation by others may facilitate the purchase of social products at lower price levels. Supporting this idea, a significantly increasing linear trend was found in the OBS group (*t*(17) = 4.56, *p* < 0.05), but not in the CON group (*t*(15) = −0.23, *p* = 0.824, Fig. 2E).

### Neuroimaging results

#### Parametric modulation analysis using the value parameters

First, we searched the MPFC for any regions engaged in encoding values of purchase decisions regardless of product types (GLM#1). We performed a group analysis of one-sample t-test on the parametric maps of all subjects (N = 34) obtained from the parametric modulation analysis using value parameters. In the subsequent analyses, we focused on these ROIs to examine the group and conditional differences as well as their interactions, all of which are orthogonal to the contrasts used to identify functional ROIs (i.e., value parameters).

**Figure 3:**
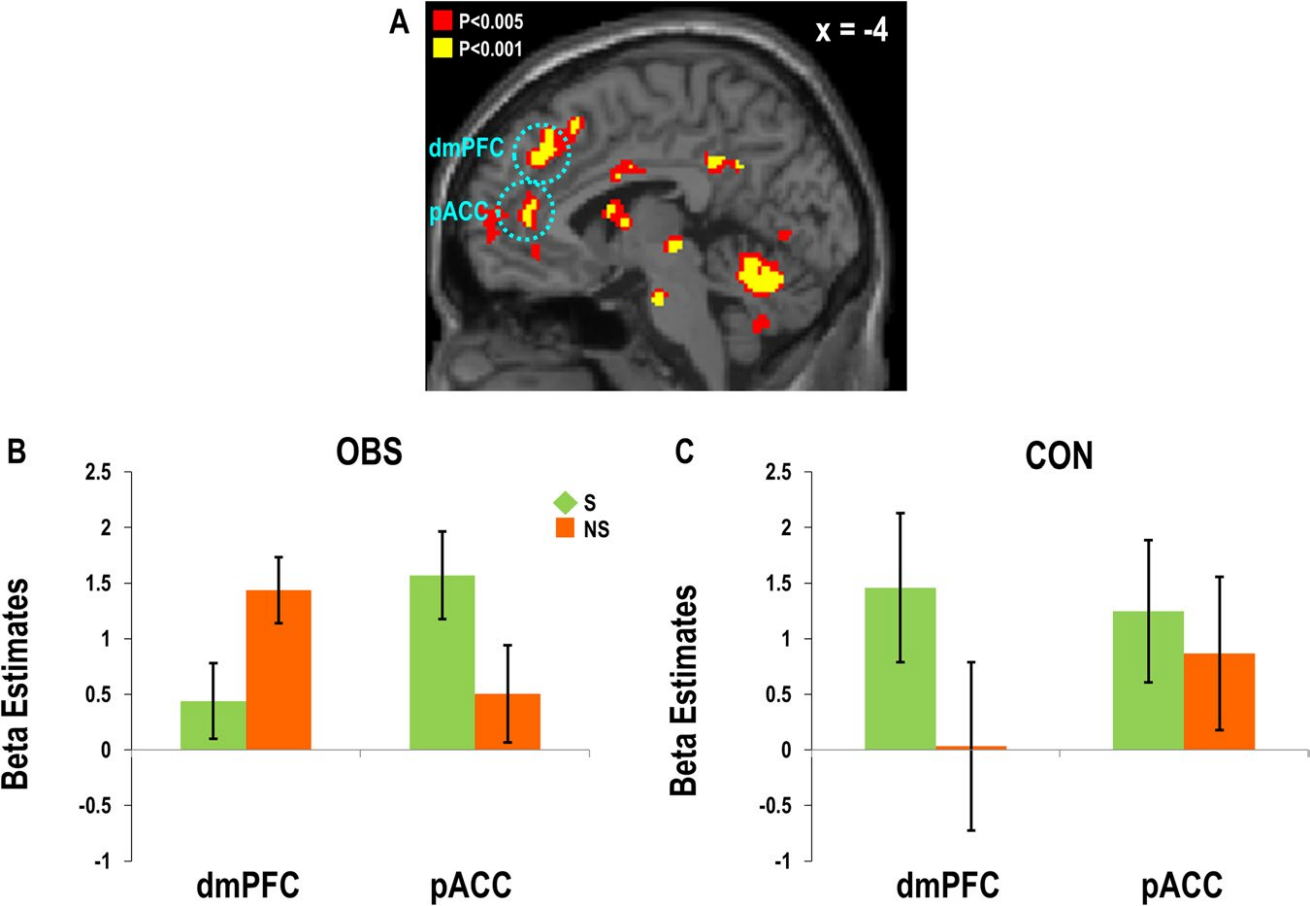
Subregions of the MPFC encoding the value parameters. (**A**) The parametric modulation analyses using individual-specific value parameters revealed value-encoding regions in the MPFC including pACC (*x* = −4, *y* = 44, *z* = 8) and dmPFC (*x* = −6, *y* = 36, *z* = 40) across all the trials in all participants. The interaction effect between brain regions and product type was found significant only in the (**B**) OBS group but not in the (**C**) CON group.

The one-sample t-test on the parametric maps of all subjects revealed that the pACC (pregenual anterior cingulate cortex: *x* = −4, *y* = 44, *z* = 8, *Z* = 3.47; all findings are reported at *p* < 0.05 small volume-corrected (SVC) unless otherwise stated) and the dmPFC (*x* = −6, *y* = 36, *z* = 40, *Z* = 3.64) showed increasing activity as the probability of purchase decision increased across both product types (Fig. 3A, See the Method section for more details about the selection of ROIs).

**Figure 4:**
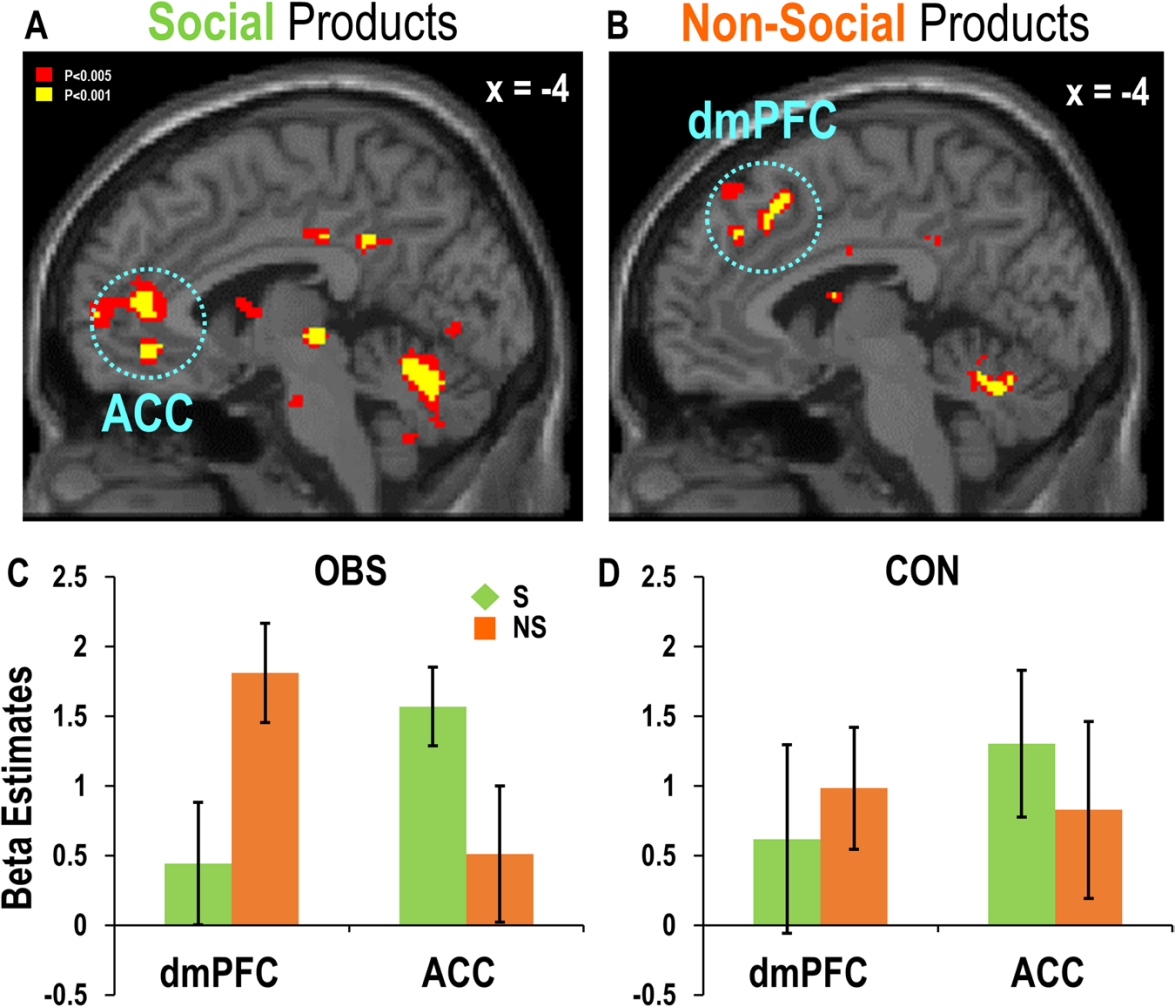
Distinctive MPFC subregions encoding the values of social versus non-social products. The parametric modulation analysis using subject-specific value parameters revealed (**A**) ventral clusters (ACC: *x* = −4, *y* = 42, *z* = −8, *Z* = 4.32; *x* = −4, *y* = 44, *z* = 8, *Z* = 3.53) for the social product condition, and (**B**) dorsal clusters (dmPFC: *x* = −2, *y* = 38, *z* = 38, *Z* = 3.28; *x* = −4, *y* = 22, *z* = 50, *Z* = 4.34) for the non-social product condition. (**C**) Greater value-related activity was observed for social (S) versus non-social (NS) product conditions in the ventral clusters and the opposite pattern in the dorsal subregions in the OBS group. (**D**) No such spatial segregation between the ventral and the dorsal MPFC subregions was found in the CON group.

Considering that social and non-social products should elicit different levels of prosocial motivation, we also expected that distinctive subregions within the MPFC would engage in encoding values of purchase decisions for social and non-social products. To test this hypothesis, we next examined value-encoding clusters in the MPFC separately for social and non-social products (GLM#2). The results showed that the ventral clusters including the sACC (subgenual anterior cingulate cortex: *x* = −4, *y* = 42, *z* = −8, *Z* = 4.32) and the pACC (*x* = −4, *y* = 44, *z* = 8, *Z* = 3.53) encoded the values of social products (Fig. 4A), whereas the dorsal clusters (*x* = −2, *y* = 38, *z* = 38, *Z* = 3.28; *x* = −4, *y* = 22, *z* = 50, *Z* = 4.34) encoded the values of non-social products (Fig. 4B), suggesting functional segregation between the ventral and dorsal subregions of the MPFC, as we hypothesized.

### Stronger MPFC functional segregation induced by observation

We hypothesized that the ACC would be involved in context-dependent prosocial valuation and the dmPFC in strategic calculation, weighing cost and benefit of purchasing social versus non-social products. Therefore, we predicted that, in the OBS condition, where purchasing non-social products may threaten the reputation of the participant, the functional segregation between the MPFC subregions in computing values of purchasing social and non-social products would increase.

First, we examined the MPFC ROIs obtained from GLM#1, which encoded the value parameters regardless of product type, to verify the pattern of functional engagement of the MPFC subregions separately for each group. Importantly, the three-way interaction of group, product type, and region was significant (*F*(1,32) = 5.16, *p* < 0.05). Supporting our hypothesis, post-hoc analyses on the three-way interaction effect showed that the two-way interaction effect between product type and region was significant only in the OBS group (*F*(1,17) = 9.47, *p* < 0.01), but not in the CON group (*F*(1,17) = 0.71, *p* = 0.41,Fig. 3B,C**)**. Next, we tested the same hypothesis using the ventral and dorsal clusters in the MPFC obtained from GLM#2, which were responsive to social and non-social product valuation, respectively. This analysis also revealed a significant interaction effect between product type and region in the OBS group (*F*(1,17) = 15.16, *p* < 0.005, Fig. 4C), showing greater value-related activity for social versus non-social trials in the ventral subregions and the opposite pattern in the dorsal subregions. No significant interaction effect was found in the CON group (*F*(1,15) = 2.82, *p* = 0.35, Fig. 4D).

### Functional connectivity of MPFC subregions

**Figure 5:**
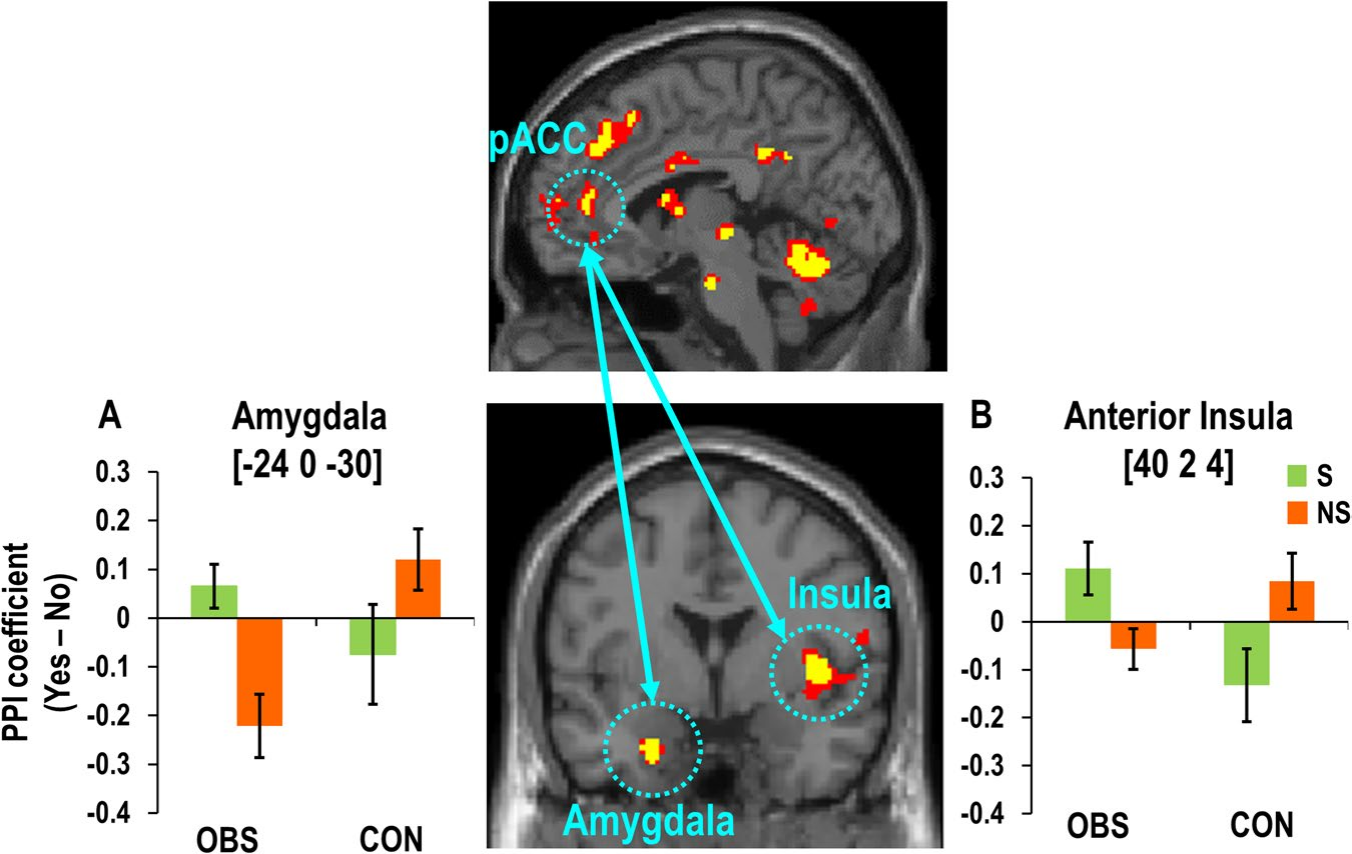
pACC functionally coupled with amygdala and anterior insula under observation. The pACC (*x* = −4, *y* = 44, *z* = 8) activity encoding decision values of social products was functionally coupled with (**A**) the left anterior amygdala (*x* = −24, *y* = 0, *z* = −30) and (**B**) and the right anterior insula (*x* = 40, *y* = 2, *z* = 4; z = 4.25) when participants purchased (vs. non-purchased) social versus non-social products in the OBS group compared to the CON group.

Based on previous findings of the intimate functional coupling between the ACC and subcortical structures such as the amygdala^41^ and the insula^42^, we next searched for the brain structures functionally coupled with the MPFC clusters encoding decision values. This analysis revealed that the pACC (*x* = −4, *y* = 44, *z* = 8) showed increased functional coupling with the left anterior amygdala (*x* = −24, *y* = 0, *z* = −30, *Z* = 4.54) and the right anterior insula (*x* = 40, *y* = 2, *z* = 4; *z* = 4.25, *Z* = 4.26) when participants purchased social versus non-social products in the OBS compared to the CON group (Fig. 5). No other seed regions showed significant PPI connectivity with other brain areas (See the Supplementary table for the list of brain regions).

### vmPFC activity associated with ethical consumption tendency in both groups

**Figure 6:**
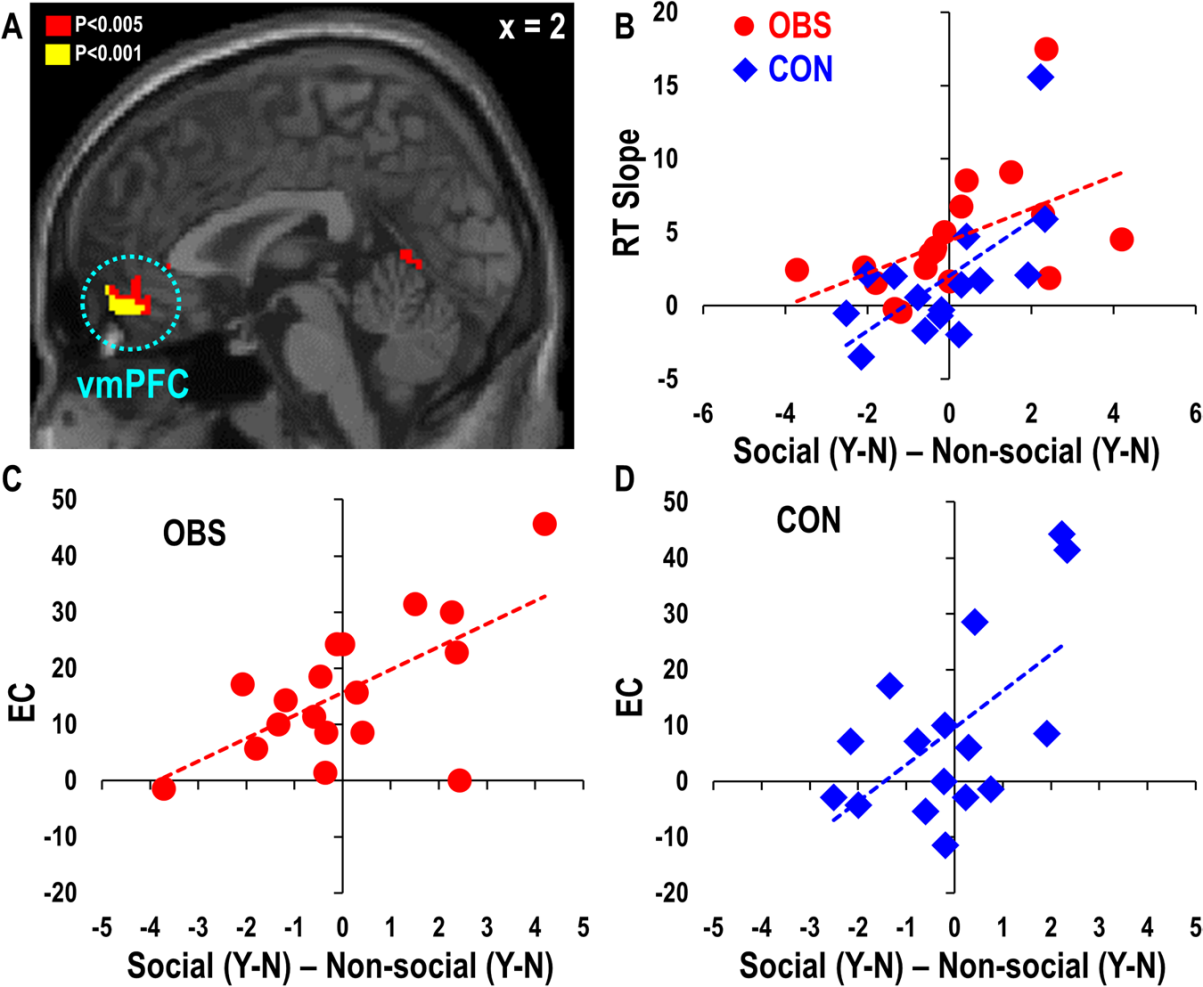
vmPFC activity encoding internalized prosocial valuation. (**A**) The vmPFC (*x* = 2, *y = *56, *z* = −14) activity encoding ethical decision bias (i.e., social products [purchase (Y) − non-purchase (N)] versus non-social products [purchase (Y) − non-purchase (N)]) showed a significant positive correlation with individual differences in ethical consumption (EC) tendency in both the (**C**) OBS and the (**D**) CON group. (**B**) The vmPFC activity predicted the degree to which the RT difference between social and non-social product condition trials varied with price range, which was significant in both the OBS and the CON group.

Given that the functional segregation within the MPFC predicted individual differences in ethical consumption (EC) in the OBS group, we further investigated the sources of such individual differences, with a special interest in revealing brain regions predicting prosocial tendencies regardless of social observation. In search of neural substrates predicting individual differences in prosocial tendencies, we regressed individual contrast maps of social products [purchase - non-purchase] versus non-social products [purchase - non-purchase] at the price/decision events against individuals’ EC and the categorical variable of group. This revealed activity in the vmPFC (*x* = 2, *y = *56, *z* = −14, *Z* = 3.79, Fig. 6A) showing a significant positive correlation with the regressor of EC (*r* = 0.59, *p* < 0.001). Post-hoc analyses revealed that vmPFC activity predicted individual differences in EC in both the OBS (vmPFC: *r* = 0.64, *p* < 0.01, Fig. 6C) and the CON (vmPFC: *r* = 0.59, *p* < 0.05, Fig. 6D) group.

Social products could activate internalized prosocial values in both groups. Such internalized prosocial values could facilitate decisions to purchase social products at a low price but slow down decisions not to purchase social products at a high price due to the increasing conflict with economic value maximizing motivation. Thus, an increase in reaction time as a function of price level for social products should correlate with the strength of internalized prosocial values, that is, the degree to which vmPFC activity correlates with the value parameters of the social product. To test this hypothesis, we computed subject-specific RT gradient scores, which show the linear trend of changes in RT differences between social and non-social products as a function of price level. We excluded one participant’s RT slope data as an outlier exceeding three standard deviations, based on the average RT slope. Confirming our hypothesis, we found a significant positive correlation between the RT gradient scores and vmPFC activity (*r* = 0.54, *p* < 0.005), which remained significant when tested separately in the OBS (*r* = 0.51, *p* < 0.05) and the CON (*r* = 0.63, *p* < 0.05) group (Fig. 6B). Taken together, these findings suggest that the vmPFC may be involved in internalized (or dispositional, subject-specific) valuation of prosocial choices, regardless of price information and the presence of observers.

### Neural responses to social versus non-social products at the time of decision

We also examined differential neural responses to social versus non-social products, regardless of value encoding (GLM#3). A simple contrast of social versus non-social product condition at the time of decision event revealed the bilateral amygdala (*x* = −20, *y* = −4, *z* = −18, *Z* = 4.04; *x* = 16, *y* = −2, *z* = −16, *Z* = 4.03). In addition, we searched for brain regions showing group differences in their responses to social versus non-social products at the time of decision (see Table S1).

### Neural responses to the brand logos of social versus non-social products

A simple contrast of social versus non-social product condition at the time of brand logo presentation (GLM#3) revealed the left nucleus accumbens (*x* = −16, *y* = 10, *z* = 0, *Z* = 4.34), right caudate nucleus (*x* = 12, *y* = 10, *z* = 8, *Z* = 3.65).

### Effect of choice difficulty

Given a recent finding that choice difficulty can be negatively associated with vmPFC and positively associated with dmPFC activity^43^ we also examined the effect of choice difficulty by modulating the regressor of the decision event with the parameter of reaction time on a trial-by-trial basis. All MPFC clusters encoding values of social and non-social products remained unchanged even after controlling for choice difficulty (Fig. S1). These results further support the selective involvement of the MPFC in encoding decision values.

## Discussion

The present study investigated the neural mechanism of observer effects on prosocial decision-making, using a consumer decision task. Consistent with previous studies^8,12,44^, participants in the OBS group, compared to the CON group, showed significantly higher purchase rates at the medium price level and faster decision times for social than non-social products at lower price levels. Most importantly, fMRI results revealed spatially segregated activation patterns between the ventral (i.e., ACC) and dorsal (i.e., dmPFC) subregions within the MPFC, encoding values of decision for social and non-social products, respectively, only in the OBS group. This observation-induced functional segregation between the ACC and the dmPFC also predicted individual differences in prosocial behavior only in the OBS group, but not in the CON group. In addition, the pACC showed strong functional coupling with the amygdala and the anterior insula during decisions to purchase social versus non-social products. Finally, replicating previous findings on its involvement in prosocial valuation, an increase in vmPFC activity was associated with greater biases toward ethical consumer decisions and predicted price-related increases in reaction times in both the OBS and the CON group. Taken together, the present findings suggest that anatomically segregated subregions along the axis of the ventral-to-dorsal MPFC may be differentially involved in computing values of prosocial decisions under observation by others.

The ACC preferentially encoded the values of social versus non-social products, showing a significant functional coupling with the anterior ventral amygdala and the anterior insula during the choice of social versus non-social products (i.e., the pACC) and predicting individual differences in prosocial behavior (i.e., the sACC), only in the OBS group. The ACC, which is known to be implicated in self-referential processing^45,46^, has been proven particularly sensitive to social observation or evaluation by others^14,15^. This region has also been associated with increased prosocial decisions in the presence of peers^17^, consistent with its role in the observer effect and reputation management^16^, and has been recently shown to compute social values in a context-dependent manner^47^. In addition, similar to those under social pressure due to observation by others, people who were explicitly asked to make donation decisions using money endowed by the experimenter showed increased pACC activity and prosocial behavior^48^. Our findings confirm and further elaborate this idea by showing that this region contributes to reputation management by computing values of context-dependent socially desirable behavior.

The ACC is also known to play a key role in regulating emotional conflict detected by the amygdala^49^, possibly via its intimate functional connectivity with the amygdala^41^ as well as the anterior insula^42^. The present findings suggest that the purchasing action of social versus non-social products promoted by the ACC may be an active process of regulating the signals arising from the ventral amygdala and the anterior insula, which may reflect emotional/motivational conflicts caused by increased reputational concerns under observation by others.

Replicating previous findings, we found that vmPFC activity predicted individual differences in prosocial tendencies especially when deciding to purchase social versus non-social products. This effect was identified in both groups of participants, highlighting the role of the vmPFC region in internalized prosocial valuation. Revealing its internalized nature of valuation, vmPFC activity also predicted individual differences in the degree to which RT differences between social and non-social products changed linearly as a function of price level in both groups; this might be a behavioral indicator of internalized prosocial valuation in conflict with increasing price level of social products with respect to non-social products. The present findings suggest that the increased vmPFC activity may elicit dominant response repertoires, which are likely to be socially desirable or prosocial choices in social situations. It should be noted, however, that vmPFC is not exclusively involved in intuitive/automatic valuation process, and the distinction of intuitive vs. controlled processes is a matter of degree.

The vmPFC activity predicting individual differences in ethical consumption tendency, regardless of observation manipulation, is incompatible with some previous studies reporting context-modulated valuation process encoded by vmPFC activity. For example, value-related activity of vmPFC was shown to be modulated by emotional priming for judgment^50^ and instructional cues for food choice^51^, craving regulation^52^, or financial decision^53^. One possible way of resolving this conflict would be to assume that the valuation process of vmPFC may be differentially modulated by specific context. That is, vmPFC valuation process can be context-dependent when the context intuitively biases the direction of decision without causing explicit conflict. In contrast, in this experiment, the observation context may have indiscriminately promoted competition between incompatible values that could be detected and regulated by pACC, rather than vmPFC. Future studies should investigate specific decision context that differentially modulate the valuation processes subserved by distinctive mPFC subregions.

Several studies have shown that vmPFC activity is commonly involved in decisions made for both self and others. For example, when participants were asked to estimate a stranger’s preference for movies with little prior knowledge of him/her, the common vmPFC activity for both self and others was found^29^. A post-hoc analysis revealed that such a common vmPFC activity reflected a mixture of self- and other-simulation processes, consistent with a recent report that vmPFC activity increased with egocentric bias in the estimation of others’ preferences^25^. In another study reporting common vmPFC activity for both self and other, participants were asked to estimate the temporal preferences (i.e., smaller-sooner vs. larger-later rewards) of others after being fully familiarized with the preferences of their partners through repeated practice trials. These findings, therefore, indicate that vmPFC can be involved in computing the value of choices for others, only when such valuation process is *internally* driven via familiarization with others’ preferences or through self-simulations.

In the present study, the dmPFC encoded subject-specific parameters of decision values for non-social, rather than social, products under social observation. The dmPFC has been implicated in encoding predictive information about future rewards^54–56^ as well as in mentalization or perspective-taking^21^. Combining these lines of research, it has recently been shown that the dmPFC computes values of decisions for others^24,25,38^ and responds to outcomes received by others^26–28^. Although dmPFC activity has often been associated with prosocial behavior^26,57^, the present study once again supports the idea that dmPFC activity does not necessarily contribute to prosocial valuation^38^, because the decisions to purchase social and non-social products require value computation for others and self, respectively. This conflicting role of the dmPFC in prosociality should be more carefully addressed by considering differences in experimental contexts across studies. For example, dmPFC activity may predict prosocial behavior only when the experimental task automatically triggers selfish behavioral responses while prosocial behavior is strategically more beneficial. Conversely, the same region may be engaged when the experimental context automatically triggers prosocial motivation while economic value maximization is strategically more beneficial. Given that observation not just increased the tendency of purchasing social products but also reduced the tendency to purchase non-social products, participants were likely to think that buying non-social products would lower their reputation under social observation. Therefore, it is likely that the increased dmPFC activity when buying non-social products in the observational group reflects an increased cognitive cost due to a conflict between economic value maximization and observation-induced motivation for impression management.

There are several alternative non-social account of dmPFC function, such as foraging decisions^58,59^, model-based valuation processing^60,61^, and the attentional control^62^, associated with switching between automatic and controlled processing^63^. In fact, the dmPFC cluster that encodes the values of purchasing non-social products is located closer to the cluster linked to action monitoring^16,64^ than to the area linked to mentalization^16,21^. This region has been shown to code for both positive and negative subject values^65^, which may be interpreted as reflecting arousal, saliency, and/or attentional shift. Importantly, all MPFC clusters remained significant even after controlling for reaction time as a covariate. This result demonstrates that the value-related activity in the MPFC subregions does not simply reflect choice difficulty^43^. According to the alternative interpretation, the dmPFC is engaged when there’s a need to disengage intuitive/familiar valuation system and shifting attention from familiar/internal bodily states to novel/external environment. In a similar vein, increased dmPFC activity for non-social products may elicit a switch from the ventral MPFC system, which is more internally focused and rather narrowly tuned in a socially desirable direction, to deliberate and strategic value maximization^22,40^, often associated with selfish/dishonest behavior^38,66^ in social settings.

A possible alternative account of the functional segregation between the MPFC subregions reported in the present study may come from recent literature on the internal versus external mode of valuation^23^. According to this view, the vmPFC encodes internal valuation sensitive to bodily signals like hunger and satiety^67,68^ being particularly sensitive to outcome devaluation^69,70^. Importantly, several studies have shown that vmPFC activity covaries with heart rate variability^71,72^, which is also predictive of individual differences in decision value encoding^72^. Consistent with these findings, the present study suggests that vmPFC activity may indicate the degree to which one’s value computation for prosocial behavior is internalized and therefore immune to reputational challenge elicited by social observation. In contrast to the vmPFC, the dmPFC has been shown to be driven mostly by sensory attributes of external incoming environmental stimuli^73,74^. Therefore, it can be inferred that social observation would increase a conflict between two competing values: one for seeking reputation and the other for economic value maximization for non-social products. This could lead to a switch from internal to external valuation mode, which would then serve to sample additional external sensory information such as visual properties and price information of products to search for a more appropriate choice option. In addition, increased value-related dmPFC activity for non-social products under observation may be particularly prominent when one is alternatively switching between choices for social and non-social products within the same task, because, only in such situation, one needs to disengage the internal valuation system (subserved by vmPFC) for social products and switch attention to the external valuation system (subserved by dmPFC) for non-social products.

One of the limitations in our study is that individual differences in ethical consumption behaviors may be confounded with one’s ability to pay attention to social vs. non-social product logos during the purchase task in the present study. In order to rule out this possibility, a future study may need to measure one’s baseline attention to social vs. non-social products (e.g., via eye-tracking device) unaffected by social observation.

In conclusion, present study found that social observation during a consumer decision task recruits anatomically and functionally segregated neural valuation systems differentially involved in prosocial decisions. Specifically, the vmPFC and dmPFC contribute to internalized prosocial value computation and strategic value maximization, respectively, while the ACC promotes reputation via context-dependent prosocial behavior. The present findings provide important insights into our understanding of the organizing principles of distinctive neural valuation systems, which can interact with each other to maximize one’s capacity for adjusting to challenging social contexts.

## Materials and Methods

### Participants

Forty-two participants were randomly assigned to either the observation (OBS) or the control (CON) group and performed a virtual shopping task in the scanner. We excluded data obtained from six participants who had responded to less than half of the total number of trials (possibly due to falling asleep during the task). Additionally, one male participant from the CON group was also removed due to excessive head motion (over 3 mm), and one female participant from the CON group was excluded due to abnormal behavioral data (i.e., increasing probability of purchase as the price level increased for the same product). A total of 34 participants (18 in the OBS group: 11 males and 7 females; mean age = 23.83, SD = 2.28; 16 in the CON group: 11 males and 5 females; mean age = 24.62, SD = 4.62) were included in the final fMRI analyses. All experiments were performed in accordance with the relevant guidelines and regulations. The Institutional Review Board of Korea University approved the experimental procedures and all participants provided informed written consent prior to the task. All participants were paid a total of KRW 30,000 (USD 30) (KRW 26,000 for participation, plus KRW 4,000 for purchasing the products during the task).

### Stimuli

We created an image pool of four different types of food items (i.e., cookies, chocolate, bread, and Korean traditional rice cake). Food items were selected based on their popularity and affordability among college students. We prepared 20 food items whose shape, colour, and quantity were matched and further divided each set into two subsets containing 10 items each. Individual items in each subset were comparable in terms of ethical value, likability, perceived quality, and familiarity, verified by ratings obtained from a separate group of participants in a pilot study (N = 11). The Becker-Degroot-Marschak (BDM) method^75–77^ was used to estimate the optimal price of each item, where participants in the same pilot study reported their willingness to pay for each product while bidding against a computer agent.

We did not collect idiosyncratic preferences of the items without the logos and prices prior to the main task in the fMRI study, because, in such a case, participants may choose to maintain consistency with the previously reported preferences, and such a motivation for decision consistency may then lead to diminished ethical consumption biases during the main task.

Each set was labelled as either social- or non-social products, which was in turn indicated by unique logo images presented at the logo/item display events on the upper left-hand corner of the food item picture (Fig. 1). The paring between the stimuli sets and product type was counterbalanced across participants. We informed participants that the social versus non-social products were produced by social versus conventional enterprises, which differ in their degree to which social impact was valued over purely commercial profit. Because we focused on the difference in social values between products of social and conventional enterprises, we referred to these items as social and non-social products, respectively, throughout the study.

### Task and procedures

In a novel “ethical consumption task”, participants were instructed to make a series of binary decisions on whether or not to buy each food item at a given price. All participants in this study received detailed information about social enterprises before starting the experiment such that all of them experienced social pressure toward ethical consumption, which was a necessary manipulation for the purpose of the study. However, unlike previous studies using a within-subjects design in which each participant was exposed to both observation and control conditions, we manipulated observation across participants to minimize the risk of demand characteristics and/or carry-over effects, while exposing participants to both reputation-sensitive (i.e., decision to purchase social products) and control (i.e., decision to purchase non-social products) conditions. Each participant viewed each food items 7 times across 7 price levels (25%, 50%, 75%, 100%, 125%, 150%, 175%) in a single functional scan run, which included 70 social and 70 non-social product condition trials (140 trials in total). A single trial consisted of a fixation period (1–3 s), a logo/item event where an image of a food item and a company brand logo were shown to indicate the product type as either social or non-social (2–4 s), and a price/decision event where a price was presented and participants were prompted to make a decision. The order of the items, product types, and price levels was determined in a pseudo-random manner such that social and non-social product trials alternated throughout each run and any large difference in price level between two consecutive trials was avoided.

In the present behavioral task, we used a binary choice task (i.e., yes/no) rather than a 4-point preference rating task (i.e., “strong no”, “strong no”, “strong no”, and “strong no”), as used in previous food evaluation task^51^, in order to create a behavioral task that is as similar as possible to the actual purchase situation in real life. To establish credibility of the experimental task, participants were told prior to the experiment that they would be asked to actually purchase one of the food items they decide to buy during the task. Upon completing the task, each participant received one of the products they decided to purchase during the task. The specific type of food item and its corresponding price were randomly drawn from the participants’ actual decisions. All participants were presented with an open question asking about the purpose of the overall experiment after the experiment, and none of the participants successfully reported the real experimental purpose in this open question.

### Neuroimaging procedures

#### FMRI data acquisition

We acquired the entire neuroimaging data using a Siemens Magnetom Trio, a 3 T Trim system with a 12-channel head matrix coil located at the Korea University Brain Imaging Center. T2*-weighted functional images were obtained using gradient-echo echo-planar pulse sequences (TR = 2000 ms; TE = 30 ms; FA = 90°; FOV = 220 mm; 78 × 78 matrix; 36 slices; voxel size = 2.8 × 2.8 × 3.0 mm^3^). The stimuli were presented via an MR-compatible LCD monitor mounted on a head coil (refresh rate: 85 Hz; display resolution: 800 × 600 pixels; viewing angle: 30° horizontal, 23° vertical). Participants used two buttons of a four-button MR-compatible response grip during the experiment. Each functional run lasted about 15 min.

### Pre-processing procedures

FMRI data were preprocessed and analysed using Statistical Parametric Mapping 8 (SPM8). All functional images were corrected for slice timing and head motion, normalized to the Montreal Neurological Institute (MNI) echo-planar imaging (EPI) template, resampled at a voxel resolution of 2 × 2 × 2 mm^3^, and spatially smoothed by using a Gaussian filter with 6-mm FWHM (Full-with-half-maximum).

### GLM#1: Model-based parametric modulation analysis with decision value parameters for all products

We conducted a parametric modulation analysis to identify brain regions that encode trial-by-trial fluctuations of decision values for both types of products, similar to previous studies^24,78^. First, we fitted each individuals’ binary decision data for social or non-social products to a sigmoid function to estimate participant-specific probability curves of purchasing social or non-social products as a function of the seven inversely coded price levels. In Equation (1) shown below, the variable *x* denotes the price level of each product, *f(x*_*i*_) is the probability of purchasing the product in trial *i*, and parameter *a* and *b* indicate the slope of the sigmoid function and the offset criterion, respectively.

1 $$f({x}_{i})=\frac{1}{{e}^{a(b-{x}_{i})}+1}$$

To identify brain regions encoding value parameters regardless of the product type (social and non-social products), regressors of social and non-social product trials were combined. We modelled the events of the logo/item and price/decision separately, and added subject-specific decision value parameters combined for both product trials to the regressors of the price/decision event. All button-press events and six motion regressors were additionally modelled as covariates.

### GLM#2: Model-based parametric modulation analysis with decision value parameters for social and non-social products

GLM#2 was identical to GLM#1, except that the regressors of social and non-social product trials were separated and two decision value parameters were added to the regressor of the price/decision events for the corresponding product trials.

### GLM#3: Basic GLM analysis

Preprocessed data were analysed by using a general linear model (GLM), which included eight regressors of interest: the logo/item events and the price/decision events with different types of products (social or non-social) and choices (purchase or non-purchase). The button-press events were added to the GLM as a regressor to reduce any noise associated with pressing the button. Six additional covariates of the realignment parameters (x, y, and z translations and pitch, roll, and yaw rotations) were included as motion regressors in order to capture any movement-related artifacts. Contrast images of social versus non-social products and the interaction between product type and choice during the logo/item event or the price/decision event were generated for each participant. The individual contrast images were subjected to two-sample t-tests for group comparison.

### Voxel-wise multiple regression analyses

Each participant’s behavioral index of ethical consumption (EC) tendency was calculated by subtracting the probability of purchasing non-social (*NS*_*i*_) products from that of purchasing social (*S*_*i*_) products at the *i*-th price level and averaging across all seven price levels, as shown in Equation (2) below.

2 $$EC=\frac{{\sum }_{i=1}^{7}({S}_{i}-N{S}_{i})}{7}$$

Individual contrast maps of social products [purchase - non-purchase] versus non-social products [purchase - non-purchase] at the price/decision events were regressed against the interaction variable of the individuals’ EC and the categorical variable of group, by computing the following whole-brain second-level multiple regression model in Equation (3) below:

3 $${y}_{N}={\beta }_{G}{x}_{G}+{\beta }_{B}{x}_{B}+{\beta }_{Int}({x}_{G}\times {x}_{B})+\varepsilon $$

where *x*_*B*_, *x*_*G*_, and *y*_*N*_ indicate the individual participants’ EC scores, the group variable (i.e., OBS group = +1, CON group = −1), and the neural index (i.e., the individuals’ contrast maps of social products [purchase − non-purchase] versus non-social products [purchase − non-purchase]), respectively.

### Examining the functional segregation between the MPFC subregions

We quantitatively measured the degree to which distinctive clusters encoding value parameters within the MPFC are functionally segregated. We calculated the mean of the ventral clusters (i.e., ACC) encoding the values of the social products and did the same for the dorsal clusters (i.e., dmPFC) encoding the values of the non-social products, which were obtained from the GLM#2, for a direct comparison between product types within and between the two regions.

### Psychophysiological interaction (PPI) analysis

To identify brain regions showing functional connectivity with the MPFC subregions encoding values obtained from the GLM#1 and GLM#2, we performed psychophysiological interaction (PPI) analyses. We generated the PPI variables by extracting time series data from the seed regions in each participant and using the interaction contrast (social [purchase − non - purchase] versus non-social [purchase − non-purchase]). Two-sample t-tests for group comparison were performed on the resulting individual PPI parametric maps.

### Statistical thresholds

Given our *a priori* anatomical hypothesis, we applied small volume corrections (SVC) for multiple comparisons, and restricted the search volumes to three MPFC subregions (spheres with radii of 15 mm) of interests (*x* = 0, *y* = 56, *z* = 2; *x* = 8, *y* = 54, *z* = 8; *x* = 9, *y* = 38, *z* = 43) that have been implicated in other-regarding decision-making in previous studies^38,79^. We also added the bilateral striatum (left: *x* = −8, *y* = 6, *z* = 6; right: *x* = 10, *y* = 6, *z* = 14; 15-mm radius spheres), the amygdala (left: *x* = 18, *y* = 2, *z* = −16; 15-mm radius sphere), and the insula (left: *x* = 32, *y* = 20, *z* = −8; 20-mm radius sphere), which have been reported to be responsive to observation by others^14^, to the search volumes. The coordinates above were also mirrored for search volumes in the opposite hemispheres.

To avoid false negatives, we also report all clusters passing the threshold of *p* < 0.001 (uncorrected) with a cluster size of 10 voxels (Table S1). MNI coordinates were transformed to Talairach space using nonlinear transformation^80^, to find the labels of corresponding brain regions.

## Electronic supplementary material

Supplementary Information
