# Supplementary material for: Social Observation Increases Functional Segregation between MPFC Subregions Predicting Prosocial Consumer Decisions
Source: Sci Rep. 2018 Feb 20;8:3368. doi: 10.1038/s41598-018-21449-z (PMC5820324; doi:10.1038/s41598-018-21449-z)
Supplement: Supplementary file 1 — Supplementary Information [file 41598_2018_21449_MOESM1_ESM.doc]

**Social Observation Increases Functional Segregation between MPFC Subregions Predicting Prosocial Consumer Decisions**

Daehyun Jung^1,2^, Sunhae Sul^4^, Minwoo Lee^1,3^, Hackjin Kim^1,3*^

^1^Laboratory of Social and Decision Neuroscience

^2^Department of Brain and Cognitive Engineering

^3^Department of Psychology

Korea University, Seoul, Republic of Korea

^4^Department of Psychology,

Pusan National University, Republic of Korea

^*^**Correspondence:**

Hackjin Kim, Ph.D.

Department of Psychology

Korea University

145 Anam-ro, Seongbuk-gu, Seoul 136-701, Republic of Korea

hackjinkim@korea.ac.kr

**Supporting Information**

**Methods**

**Instruction**

*“The task you'll be starting soon is to decide whether to purchase the product based on the price, brand, and image of the product presented on the screen. When the task starts, images of various products on the market are displayed on the computer screen along with price information. You can decide whether you want to buy the product at that price or not.*

*At the end of the task, one of the products you have decided to purchase will be randomly selected, and you will actually purchase this product at the given price.*

*You will be paid a participation fee (KRW 26,000) by default, along with a small additional budget (KRW 4,000) to purchase products. You can only purchase products within this budget, and the amount remaining after purchase will be added to your basic purchase price of 26,000 won and will be paid to you along with your purchase. For example, if you have decided to buy a product A at 3,000 won, and this decision is randomly selected, you will receive 27,000 won (26,000 won + 4,000 won - 3,000 won) together with the product A.*

*In this assignment, four kinds of products (i.e., bread, rice cake, cookie, and chocolate) are presented. All products are divided into two types according to the type of manufacturer: one type is a product made by a general company and the other type is a product made by a social enterprise (such as a fair-trade company). Information about the manufacturer can be found in the brand logo on the top left of the product image (See image below).*

*Please make a purchase decision with consideration of product image, price, and producer information in a comprehensive manner, and make your purchase decision as soon as possible. (After 10 seconds, the system will automatically proceed to the next trial). All recorded personal information will be used for statistical analyses anonymously and will be discarded after the end of the study.*

*< Additional Instruction for the Observation Group >*

*“The current computer program is rather unstable and your response data may not be saved properly. Just in case, therefore, two researchers will be inevitably watching your experimental screen outside and record manually all of your responses (Actually showing the monitor that will be watched by the researchers)”*


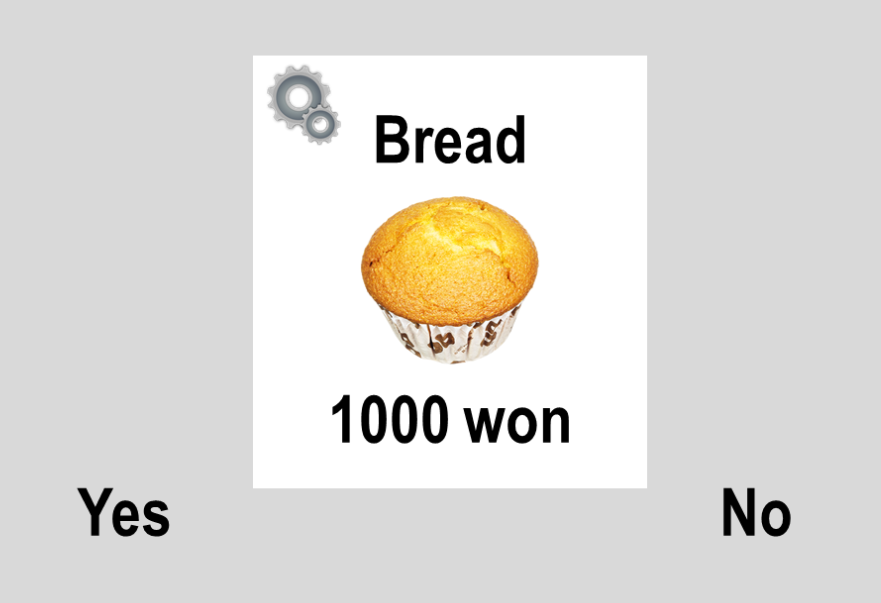

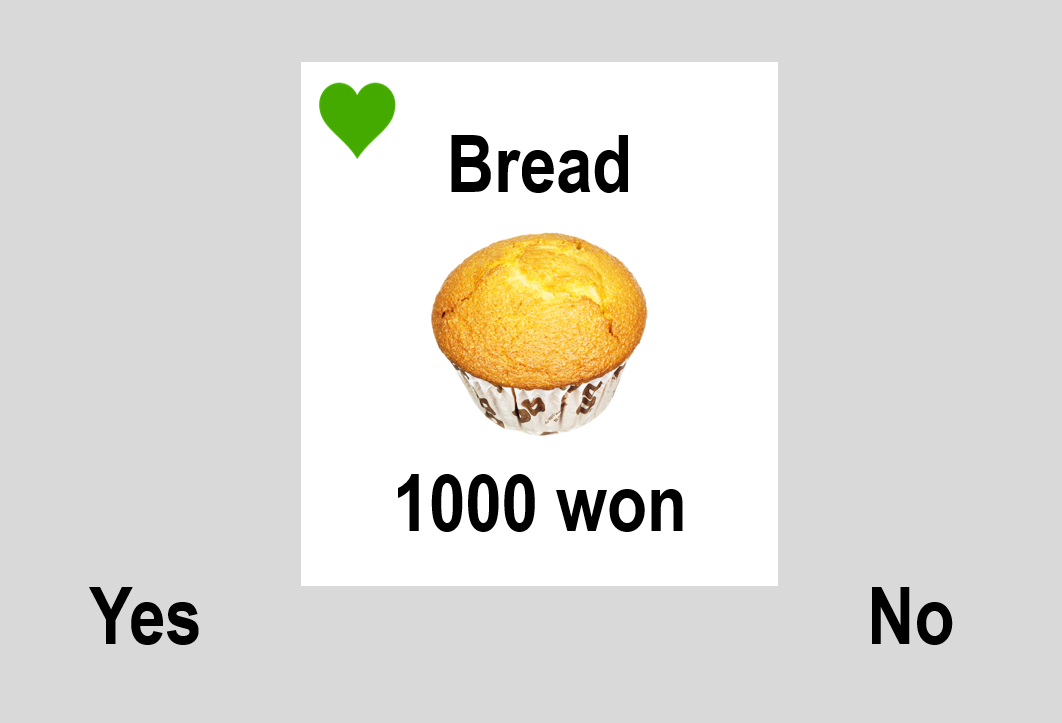


*Examples of computer screens showing the products made by conventional enterprise (left) and social enterprise (right) product made by conventional enterprise (muffin: https://cdn.pixabay.com/photo/2017/04/04/16/34/cake-2201816_960_720.jpg), brand logo of social product image (cogwheel: https://cdn.pixabay.com/photo/2013/07/12/12/30/cogwheel-145804_960_720.png) and non-social product image (green colored heart: https://cdn.pixabay.com/photo/2013/07/13/12/07/heart-159223_960_720.png) by Pixabay is licensed under CC0 Creative Commons (https://creativecommons.org/publicdomain/zero/1.0/legalcode).*

**GLM#S1: Deviation from decision value parameters for non-social products**

We conducted another parametric modulation analysis to identify brain regions that correlated with trial-by-trial fluctuations of decision bias due to product type by adopting the method used in the previous studies^1^. To estimate subject-specific decision bias parameters, we first fitted each individual’s binary decision data for non-social product to a sigmoid function to estimate participant-specific probability curves of purchasing non-social product as a function of inversely coded seven price levels (Equation 1). Next, we calculated the model estimation error (MEE), that is, the deviation from value parameters of non-social products. For each trial, the MEE is derived by subtracting the estimated decision probability *f(xi)* for non-social product trials from the actual purchase decisions *Di* for social product trials (Equation S1).


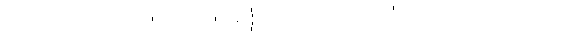


We separately modeled 4 regressors based on the type of product (social or non-social) for the logo/item event and price/decision event and 2 MEE parameters were added to the regressors of the price/decision event for both types of product. All the button press events and six motion regressors were additionally modeled as covariates.

**Table S1.** List of all brain structures identified at the significance level of *p* < 0.001 (uncorrected) and an extent threshold of 10 voxels. * indicates the brain regions still survived at significance level of *p* < 0.05 (FDR corrected at cluster level)

| Brain region | *Z* | *x y z*  (Peak in MNI space) | | |
| --- | --- | --- | --- | --- |
|  | | | | |
| **Regions correlated with the parameter of decision values** | | | | |
| ***Product (price/decision events, GLM#1)*** | | | | |
| pACC | 3.47 | -4 | 44 | 8 |
| dmPFC | 3.64 | -6 | 36 | 40 |
| Angular Gyrus (L) | 3.83 | -58 | -64 | 24 |
| Anterior Cingulate Cortex | 4.39 | 0 | -2 | 30 |
| Cerebellum (R) | 4.01 | 48 | -64 | -42 |
| Hippocampus (R) | 3.45 | 16 | -4 | -14 |
| Inferior Frontal Gyrus (L)* | 4.03 | -48 | 46 | 12 |
| Inferior Frontal Gyrus (R) | 3.35 | 30 | 26 | -8 |
| Inferior Temporal Gyrus (R) | 3.70 | 46 | 2 | -40 |
| Insula (R) | 3.50 | 44 | 6 | 4 |
| Middle Cingulate Cortex | 3.79 | 6 | -16 | 30 |
| Middle Frontal Gyrus (L)* | 4.31 | -22 | 28 | 56 |
|  | 4.30 | -34 | 6 | 36 |
| ParaHippocampal Gyrus (L) | 4.65 | -10 | 0 | -20 |
| Postcentral Gyrus (R) | 5.27 | 48 | -22 | 42 |
| Superior Frontal Gyrus (L) | 3.75 | -18 | 44 | 42 |
| Superior Frontal Gyrus (R) | 3.65 | 18 | 40 | 48 |
| Superior Medial Gyrus (L) | 3.73 | -4 | -40 | 34 |
| Superior Temporal Gyrus (R) | 3.78 | 60 | -56 | 20 |
| SupraMarginal Gyrus (R) | 3.78 | 48 | -44 | 30 |
| Thalamus (R)* | 4.63 | 10 | -16 | -2 |
|  |  |  |  |  |
| ***Social > Non-social products (price/decision events, GLM#2)*** | | | | |
| sACC | 3.47 | -4 | 42 | -8 |
| pACC | 3.53 | -4 | 44 | 8 |
| Cerebellum (L)* | 5.43 | -16 | -54 | -22 |
|  | 4.00 | -30 | -32 | -30 |
| Hippocampus (R) | 3.46 | 20 | -6 | -12 |
| Middle Cingulate Cortex | 3.93 | 8 | -2 | 42 |
| Middle Temporal Gyrus (L) | 3.86 | -62 | -16 | -20 |
| Olfactory Cortex (R) | 4.24 | 12 | 10 | -16 |
| ParaHippocampal Gyrus (L) | 3.54 | -16 | 2 | -16 |
| Postcentral Gyrus (R) | 5.04 | 42 | -26 | 42 |
| Putamen (R) | 3.72 | 32 | -12 | -4 |
| Cerebellum (R) | 3.71 | 24 | -44 | -44 |
| Superior Frontal Gyrus (L) | 3.99 | -18 | 42 | 36 |
| Superior Medial Gyrus (L) | 3.73 | -12 | 58 | 8 |
| Thalamus (R)* | 4.61 | 10 | -18 | 0 |
|  |  |  |  |  |
| ***Non-social > Social products (price/decision events, GLM#2)*** | | | | |
| dmPFC | 4.34 | -4 | 22 | 50 |
|  | 3.28 | -2 | 38 | 38 |
| Cerebellum (L)* | 5.20 | -10 | -54 | -18 |
| Inferior Frontal Gyrus (L)* | 4.68 | -44 | 48 | -12 |
| Inferior Frontal Gyrus (R) | 3.62 | 42 | 24 | -10 |
| Middle Frontal Gyrus (L)* | 4.10 | -40 | 22 | 50 |
| Precentral Gyrus (L) | 3.67 | -36 | 4 | 38 |
| Superior Frontal Gyrus (R) | 4.01 | 18 | 34 | 54 |
| Thalamus (R) | 3.73 | 14 | -14 | 6 |
|  |  |  |  |  |
| **Regions correlated with individual differences in prosocial decision** | | | | |
| ***Multiple regression with EC (GLM#4)*** | | | | |
| vmPFC | 3.79 | 2 | 56 | -14 |
| Nucleus Accumbens | 3.52 | -16 | 20 | -2 |
| Anterior Cingulate Cortex | 3.57 | 8 | 34 | 2 |
| Cerebellum (L) | 4.33 | -16 | -60 | -48 |
| Inferior Temporal Gyrus (L) | 3.72 | -40 | 0 | -32 |
| ParaHippocampal Gyrus (R) | 3.75 | 24 | -14 | -30 |
| Putamen (L) | 3.69 | -20 | 14 | -10 |
| Superior Frontal Gyrus (L) | 3.83 | -16 | 30 | 36 |
|  |  |  |  |  |
| **Functional connectivity (PPI) analysis** | | | | |
| ***pACC as a seed point (GLM#4)*** | | | | |
| Anterior amygdala (L) | 4.54 | -24 | 0 | -30 |
| Anterior insula (R) | 4.25 | 40 | 2 | 4 |
| Anterior Cingulate Cortex | 3.87 | -6 | 26 | 18 |
| Cerebellum (L) | 4.03 | -4 | -70 | -34 |
| Cerebellum (R) | 4.39 | 12 | -50 | -38 |
| Fusiform Gyrus (L) | 4.46 | -30 | -70 | -8 |
| Insula (L) | 3.71 | -42 | -12 | 10 |
| Medial Temporal Pole (R) | 3.75 | 40 | 8 | -36 |
| Middle Occipital Gyrus (L) | 3.61 | -16 | -98 | 10 |
| Middle Temporal Gyrus (L) | 3.88 | -48 | -66 | 8 |
| Postcentral Gyrus (L) | 3.70 | -58 | -14 | 18 |
|  |  |  |  |  |
| ***dmPFC as a seed point (GLM#4)*** | | | | |
| Anterior thalamus (R) | 3.37 | 12 | -4 | 0 |
| Insula (R) | 3.52 | 44 | 22 | -4 |
| Pallidum (R) | 3.63 | 16 | 0 | -6 |
| Inferior Frontal Gyrus (L) | 4.21 | -38 | 18 | 6 |
|  |  |  |  |  |
| **Regions correlated with the parameters of the model estimation error (MEE)** | | | | |
| ***Positive correlation w/ MEE (GLM#3)*** | | | | |
| Caudate | 3.84 | 8 | 12 | 8 |
| Inferior Frontal Gyrus (L) | 3.53 | -52 | 22 | 10 |
| Middle Frontal Gyrus (R) | 3.88 | 38 | 16 | 36 |
| Postcentral Gyrus (R) | 3.68 | 66 | -8 | 16 |
|  |  |  |  |  |
| **Analysis of Simple Contrasts** | | | | |
| ***Social > Non-social (price/decision events, GLM#4)*** | | | | |
| Amygdala (L) | 4.14 | -20 | -4 | -18 |
| Amygdala (R) | 3.76 | 16 | -2 | -16 |
| frontopolar cortex | 3.73 | 8 | 66 | 8 |
| precuneus | 3.74 | -18 | -44 | 34 |
| temporo-parietal cortex | 3.84 | 60 | -52 | 36 |
| Angular Gyrus (R) | 3.90 | 60 | -52 | 36 |
|  | 3.52 | 56 | -62 | 24 |
| Anterior Cingulate Cortex (R) | 3.56 | 4 | 38 | 26 |
| Cerebellar Vermis (R) | 4.02 | 8 | -58 | -34 |
| Hippocampus (R) | 3.89 | 16 | -12 | -16 |
|  | 3.49 | 30 | -18 | -18 |
| Inferior Frontal Gyrus (L) | 3.93 | -30 | 16 | -24 |
| Inferior Frontal Gyrus (R) | 3.44 | 52 | 40 | -8 |
| Insula (L) | 3.37 | -34 | 18 | -8 |
| Lingual Gyrus (L) | 3.35 | -4 | -78 | -4 |
| Lingual Gyrus (R) | 3.41 | 6 | -74 | -8 |
| Middle Cingulate Cortex (L) | 3.84 | -4 | -12 | 38 |
|  | 3.77 | -14 | -48 | 32 |
| Middle Temporal Gyrus (L) | 3.54 | -58 | -8 | -20 |
| Middle Temporal Gyrus (R) | 3.71 | 62 | -28 | -14 |
| Superior Medial Gyrus (L) | 3.69 | -8 | 46 | 38 |
| Superior Medial Gyrus (R) | 3.90 | 4 | 64 | 6 |
| Superior Orbital Gyrus (R) | 3.86 | 14 | 52 | -12 |
| Superior Temporal Gyrus (R) | 3.58 | 44 | -28 | -14 |
| Temporal Pole (R) | 4.60 | 40 | 18 | -30 |
|  |  |  |  |  |
| ***2 (Social vs. Non-social) X 2 (OBS vs. CON) Interaction (price/decision events, GLM#4)*** | | | | |
| Anterior thalamus (R) | 4.04 | 16 | -4 | 6 |
|  |  |  |  |  |
| ***Social > Non-social (logo/item events, both groups combined, GLM#4)*** | | | | |
| Anterior Cingulate Cortex (R) | 3.71 | 8 | 44 | 14 |
| Caudate (R) | 3.84 | 12 | 10 | 8 |
| Insula (L) | 3.85 | -42 | -4 | 8 |
| NAcc (L) | 4.41 | -16 | 10 | 0 |
| Thalamus | 4.22 | -16 | -6 | 10 |
| Cerebellum (L) | 4.21 | -32 | -42 | -26 |
|  | 3.65 | -8 | -76 | -20 |
| Cerebellum (R) | 3.33 | 14 | -54 | -16 |
| Cuneus (R) | 3.39 | 14 | -96 | 12 |
| Fusiform Gyrus (L) | 4.17 | -24 | -80 | -6 |
| Fusiform Gyrus (R) | 4.24 | 30 | -74 | -4 |
| Inferior Frontal Gyrus (R) | 3.72 | 36 | 8 | 28 |
|  | 3.82 | 48 | 30 | 24 |
| Inferior Temporal Gyrus (R)* | 4.74 | 52 | -62 | -12 |
|  | 4.46 | 48 | -38 | -22 |
| Middle Cingulate Cortex (R) | 3.47 | 10 | 24 | 36 |
| Middle Occipital Gyrus (L) | 3.96 | -42 | -82 | -2 |
| Middle Occipital Gyrus (R)* | 3.88 | 36 | -88 | 16 |
| Middle Temporal Gyrus (R) | 3.47 | 54 | -60 | 8 |
| Precentral Gyrus (R) | 4.00 | 54 | 4 | 32 |
| Superior Temporal Gyrus (R) | 3.82 | 58 | -42 | 12 |
|  |  |  |  |  |

**Table S2.** Average purchase rates and RTs per group and product condition

|  | **OBS**  **/Social Products** | **OBS**  **/Non-social Products** | **CON**  **/Social Products** | **CON**  **/Non-social Products** |
| --- | --- | --- | --- | --- |
| **Purchase rate** | **51.67 %** | **35.63 %** | **46.16 %** | **37.28%** |
| **RT** | **1197 ms** | **1196 ms** | **1222 ms** | **1236 ms** |

**Figure S1. MPFC subregions encoding the value parameters after controlling for choice difficulty**

The parametric modulation analysis using subject-specific value parameters still revealed the dmPFC (*x* = -6, *y* = 40, *z* = 34, *Z* = 3.81) and pACC (*x* = -4, *y* = 44, *z* = 12, *Z* = 3.83) clusters for both products condition even after the modulatory parameter of the reaction time on a trial-by-trial basis was added to the regressor of the price/decision events.

**
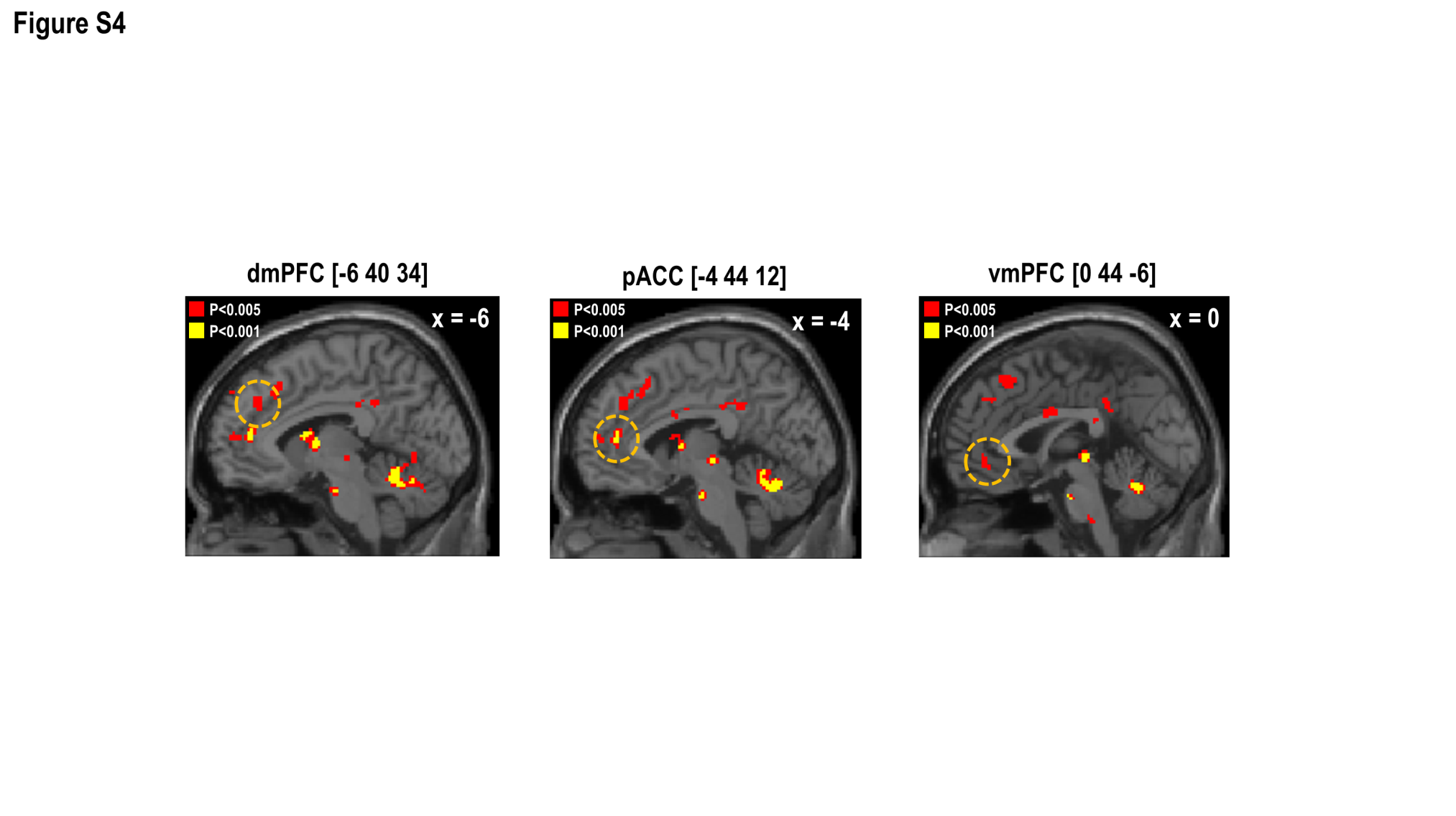
**

**Figure S2. Caudate activity signaling trial-by-trial prosocial decision bias**

Another important goal of the present study is to identify neural signals associated with the changes in purchase decision due to observation by others. Therefore, we performed a parametric modulation analysis (GLM#3) where we estimated subject-specific trial-by-trial fluctuations of neural signals related to decision bias towards buying social against non-social product (i.e., the MEE parameter, **Fig. S2a**) (See the Methods for details). The MEE parameters were significantly correlated with the right caudate nucleus activity (*x* = 8, *y* = 12, *z* = 8, *Z* = 3.84, **Fig. S2b**). A post-hoc analysis revealed that the MEE-related caudate activity was higher in the OBS group compared to the CON group (*t*(32) = 4.37, *p* < 0.001, **Fig. S2c**).

**
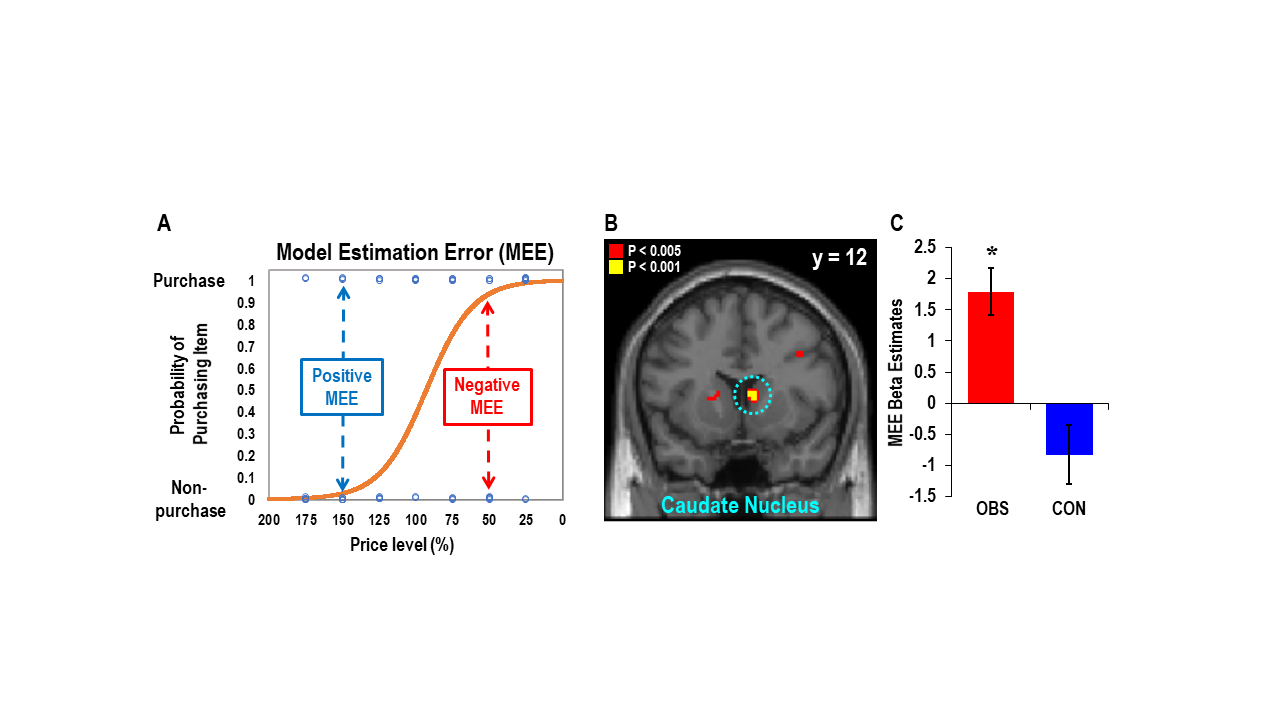
**

**Figure S3. Degree of MPFC functional segregation predicting prosocial decision**

Given the significant functional dissociation between the ventral and the dorsal MPFC clusters in the OBS group, we next investigated whether the degree of such dissociation is indeed linked to the increased prosocial purchase decision in the OBS group. We performed a correlation analysis between the degree of the MPFC functional dissociation (i.e., ventral [social – non-social] – dorsal [social – non-social]) obtained from the GLM#2 and the purchase rates for social vs. non-social products, which is, the ethical consumption tendency (EC), across participants. The result showed that the degree of the MPFC functional dissociation was significantly correlated with individual differences in EC only in the OBS group (*r* = 0.47, *p* = 0.05), and not in the CON group (*r* = 0.02, *p* = 0.95). A post-hoc analysis also revealed that the correlation in the OBS group was primarily driven by the value-encoding activity in the ventral clusters (*r* = 0.57, *p* < 0.05) rather than the dorsal ones (*r* = 0.15, *p* = 0.54). The ventral clusters can be further divided into one in the pACC and the other in the subgenual ACC (sACC), and we found a highly significant positive correlation in the sACC (*r* = 0.59, *p* < 0.05, **Fig. S3a**) in the OBS group but not in the CON group (*r* = -0.30, *p* = 2.53, **Fig. S3b**). The pACC showed only a marginally significant correlation (*r* = 0.46, *p* = 0.053) in the OBS group but not in the CON group (*r* = -0.20, *p* = 0.46).


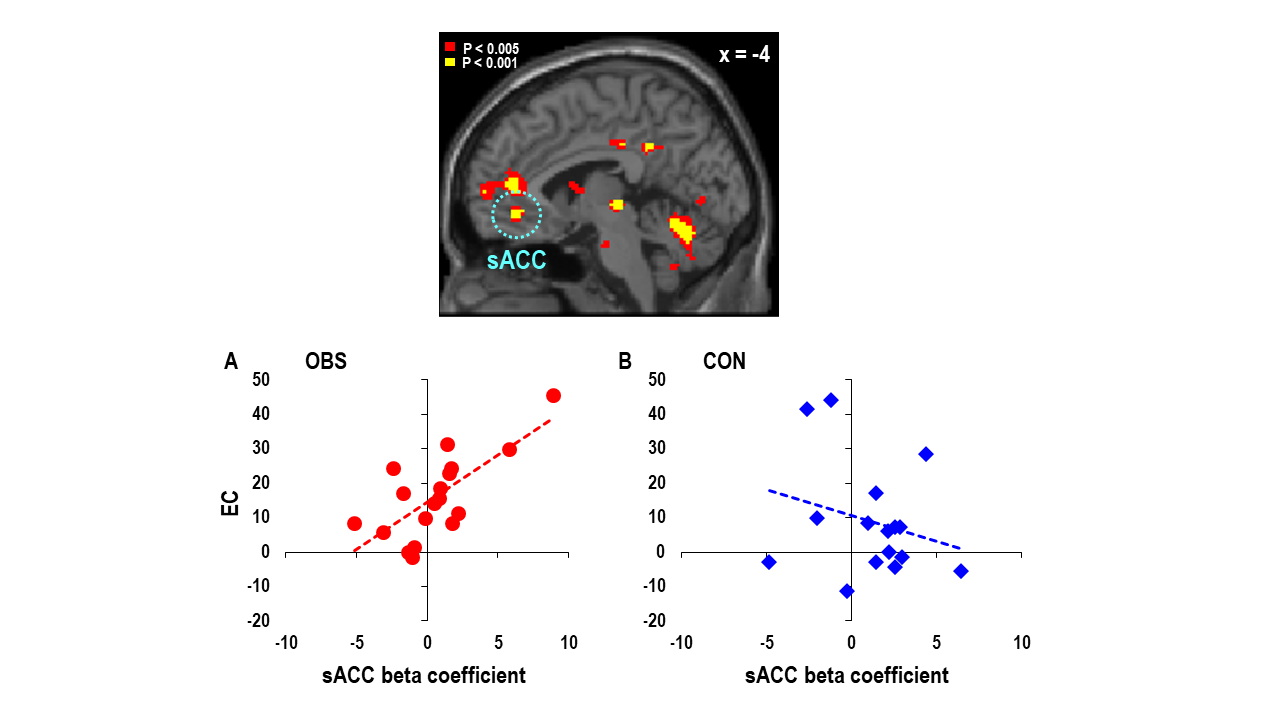


**References**

1. Kim, H., Choi, M. J., Jang I. J., Lateral OFC activity predicts decision bias due to first impressions during ultimatum games. *J Cogn Neurosci* **24**, 428-439 (2012)
